# Advanced Strategies for Food-Grade Protein Production: A New *E. coli*/Lactic Acid Bacteria Shuttle Vector for Improved Cloning and Food-Grade Expression

**DOI:** 10.3390/microorganisms7050116

**Published:** 2019-04-27

**Authors:** Marcello Tagliavia, Aldo Nicosia

**Affiliations:** 1 National Research Council-Institute for the Study of Anthropic Impacts and Sustainability in the Marine Environment (IAS-CNR), Capo Granitola, Via del mare, Campobello di Mazara (TP), 91021 Sicily, Italy; aldo.nicosia@cnr.it; 2 Department of Biological, Chemical and Pharmaceutical Sciences and Technologies (STEBICEF), University of Palermo, Viale delle Scienze, Ed.16, 90128 Palermo, Italy

**Keywords:** lactic acid bacteria (LAB), generally recognized as safe (GRAS) microorganisms, food-grade expression vectors, shuttle expression vectors, advanced food-grade cloning: flippase (FLP) recombinase, resistance cassette removal

## Abstract

Food-grade production of recombinant proteins in Gram-positive bacteria, especially in LAB (i.e., *Lactococcus*, *Lactobacillus,* and *Streptococcus*), is of great interest in the areas of recombinant enzyme production, industrial food fermentation, gene and metabolic engineering, as well as antigen delivery for oral vaccination. Food-grade expression relies on hosts generally considered as safe organisms and on clone selection not dependent on antibiotic markers, which limit the overall DNA manipulation workflow, as it can be carried out only in the expression host and not in *E. coli*. Moreover, many commercial expression vectors lack useful elements for protein purification. We constructed a “shuttle” vector containing a removable selective marker, which allows feasible cloning steps in *E. coli* and subsequent protein expression in LAB. In fact, the cassette can be easily excised from the selected recombinant plasmid, and the resulting marker-free vector transformed into the final LAB host. Further useful elements, as improved MCS, 6xHis-Tag, and thrombin cleavage site sequences were introduced. The resulting vector allows easy cloning in *E. coli*, can be quickly converted in a food-grade expression vector and harbors additional elements for improved recombinant protein purification. Overall, such features make the new vector an improved tool for food-grade expression.

## 1. Introduction

The expression of recombinant proteins in bacterial hosts other than *Escherichia coli* arouses growing interest for both protein production and delivery. In particular, in last decades many efforts have been made in order to exploit Gram-positive bacteria like lactic acid bacteria (LABs), *Bacillus subtilis* and *Bacillus megaterium*, the former group being the most widely used because of several features and advantages. In fact, both *B. subtilis* and *B. megaterium* offer very useful features, including the non-pathogenicity, the easy manipulation, the well-defined knowledge of metabolic and genetic features, along with the superior ability in protein secretion [1,2,3,4,5,6,7,8,9,10,11,12]. However, especially for food grade production, LABs are recognized as more attractive because either their biotechnological versatility and performances, and their food origin, which makes them socially more acceptable, especially for in vivo use [13].

Lactic acid bacteria (LAB) constitute a diverse group of Gram-positive microorganisms, including species of genera *Lactobacillus*, *Lactococcus*, *Streptococcus*, *Pediococcus, Leuconostoc*, and *Oenococcus*, which are exploited for various applications. Most of these bacteria, used since a long time in food processing, are considered safe for human consumption and thus possess the GRAS (generally recognized as safe) status [14].

*L. lactis* is the best-characterized species of the group, and figures as the model organism, because of its easy manipulation, its sequenced genome and the development of several genetic tools in last years. This has made *L. lactis* more suitable for biotechnological uses, ranging from the production of recombinant proteins to the expression and delivery of antigens and bioactive polypeptides to mucosal surfaces [15], and more recently as a vehicle for the delivery of DNA vaccines [16,17,18,19,20,21].

Features like high resistance to the stomach acidic environment, the ability to survive in the gastrointestinal tract without colonizing it (which limits immunotolerance), the low immunogenicity, and the lack of lipopolysaccharides in its cell wall, which eliminates the risk of endotoxin shock, make such organisms highly versatile for in vivo uses, including immunization programs [22,23].

The high performances of *L. lactis* in recombinant proteins production, both secreted and cytoplasmic, as well as surface exposed, are well known. In addition to the lack of endotoxins (as gram-positive), other relevant features include as the low endo- and exoproteolytic activity, the low propensity to form inclusion bodies, the simple fermentation, and scale-up, as well as downstream processing. Overall, these properties make such non-spore forming organism a promising cell factory for high-level proteins production and their in vivo delivery for medical uses. For this purpose, several *L. lactis* constitutive and inducible expression systems have been proposed, the former being preferable for in vivo expression [24,25,26,27,28].

The use of *L. lactis* for the production of heterologous proteins, including antigens for vaccination (for a review, see References [13]), as well as the possibility of exploiting it for in vivo delivery and supplementation of digestive enzymes like beta-galactosidase [29], is currently a promising reality. Although such strategies present several advantages over traditional administration routes, further clinical trials and a proper risks assessment to human health are still necessary.

The use of recombinant and/or genetically modified organisms raises several concerns; the major ones are related to the use of recombinant bacteria in the clinical practice and in food manufacturing are related to the dissemination of cloning plasmids and/or recombinant gene constructions.

To minimize these concerns, expression systems—called food-grade—have been developed. The distinctive feature of such vectors is the lack of antibiotic-resistance markers, so that their selection, induction, and maintenance in the host rely on the use of alternative strategies, so as to preserve the GRAS status of this bacterium [13,30,31,32].

In fact, resistance markers are routinely used for the selection and maintenance of vectors in the host for research/industrial uses; however, for legal and ethical reasons, the presence of such sequences is not acceptable in food or clinical applications, so that food-grade vectors have been developed to meet industrial demands for GRAS recombinant products.

The maintenance of food-grade vectors into the cells is achieved through selection by strategies as auxotrophic complementation, sugars utilization, resistance to heavy metals, and more recently, the use of CRISPR/Cas technology [13,31,32,33], whereas systems based on chromosomal integration have been developed especially in order to overcome the structural instability of some food-grade vectors [34,35] besides the advantages arising from the lack of episomal elements.

Noteworthy, most applications where recombinant proteins have to be used as purified molecules do not strictly require food-grade expression, although the latter makes them more acceptable especially for uses in food and, for biomedical purposes, in vivo. Instead, food grade expression is mandatory whenever the expressing recombinant organism itself has to be used, as it occurs for vaccine delivery (for a review, see Reference [13]).

In such instances, one of the most popular selection system developed in *L. lactis* is based on the plasmid-mediated restore of the lactose utilization. In particular, inactivation of *lacF*, a lactose gene utilization, can be used for both selection and maintenance of transformants [36,37,38]. The food-grade vectors pNS21 series, like pNZ2122/pNZ2123, came originally from the *L. lactis* plasmid pSH71 harboring a broad host range replicon, able to replicate in many Gram-positive bacteria, such as *Lactobacillus plantarum* and *Streptococcus thermophilus*.

The constitutive expression is ensured by the strong promoter lactococcal lactose operon (lacA), and the *lacF* gene, encoding the soluble carrier enzyme IIALac, is used as a dominant selection marker in *L. lactis* strains like NZ3000, carrying an in-frame deletion of the chromosomal *lacF* gene [37]. Lactose transformants can be screened using lactose indicator plates, even if such selection often generates slow growing colonies. Moreover, the overall strategy of food-grade cloning is not without drawbacks.

In fact, even if most of such vectors are able to replicate in both *L. lactis* and *E. coli*, in the latter host they can be neither selected nor efficiently maintained. This limitation imposes to carry out all cloning steps in the Gram-positive host, where low transformation efficiency and plasmid yield are usually experienced. However, the mandatory need of selective markers other than antibiotic resistance genes in food-grade vectors makes the overall workflow more tedious than the usual cloning in *E. coli*.

Moreover, the aforementioned vectors (pNZ series), although well designed and suitable for general purposes food grade expression, lack many useful elements routinely present in expression vectors for protein production in *E. coli* or *Saccharomyces cerevisiae*. Such elements include a compact polylinker (Multi-Cloning Site, MCS) for easy cloning with minimal additional codons from MCS sequences, 6x-His Tag for protein purification by affinity chromatography (Immobilized Metal Affinity Chromatography, IMAC), the proteases (i.e., Xa or thrombin) cleavage site for efficient purification, and removal of extra aminoacidic sequences.

Furthermore, the possibility of including removable antibiotic resistance cassettes, which would make plasmids selectable in *E. coli* without compromise the subsequent food-grade status has not been explored, despite such strategy is well established and widely employed in microorganisms for different purposes (i.e., chromosomal deletions, genes substitutions, etc.). Such a strategy would combine the required features of food-grade vectors with the versatility of the cloning procedures performed in *E. coli*, based on antibiotic selection.

Removal of integrated cassettes, including selection markers, is often achieved using the Cre-loxP system or FlippaseFlippase Recognition Target (FLP-FRT) recombinase expression [39,40,41]. The latter, much more used in bacterial systems, relies on FRT-flanked cassettes in combination with FLP, a bidirectional tyrosine recombinase derived from the 2 μm plasmid of *S. cerevisiae* [42]. The recombinase recognizes a target sequence of at least 34 bp (FRT), and catalyzes the cutting and rejoining of inversely repeated FRT sites, which leaves a single FRT sequence [43,44,45]. The most widely used protocol in *E. coli* is based on the temperature-triggered FLP expression from a temperature sensitive plasmid [46,47]. This allows the concomitant induction of FLP expression and the loss of the expressing plasmid.

Herein, we describe a new food-grade shuttle vector where the presence of the aforementioned improving elements was combined with a removable selective marker, making feasible its manipulation in *E. coli*. Resulting clones are simply switched to the food-grade status through induction of FLP recombinase, which leads to the selective marker excision.

## 2. Materials and Methods

### 2.1. Strains and Plasmids

*E. coli* K38: HfrC (λ) (from N. Zinder Laboratory, The Rockefeller University, New York, NY, USA) was used as host for plasmid maintenance and manipulation.

*L. lactis* NZ3000 (MoBiTec, Goettingen, Germany) was the Gram-positive host.

The lactococcal food-grade expression vector pNZ2122 (MoBiTec) was used as parental plasmid for subsequent modifications.

The pKD13 and pCP20 plasmids [46] (from *E. coli* Genetic Stock Center (CGSC), Yale University, New Haven, CT, USA) were maintained in *E. coli* BW25141 (*F-, Δ(araD-araB)567, ΔlacZ4787(::rrnB-3), Δ(phoB-phoR)580, λ-, galU95, ΔuidA3::pir+, recA1, endA9(del-ins)::FRT, rph-1, Δ(rhaD-rhaB)568, hsdR514*) and used as Km^R^ cassette source and for FLP recombinase expression, respectively.

*E. coli* strains were grown onto LB Agar plates or LB broth, supplemented with antibiotics when needed (Kanamycin 50 µg/mL; Ampicillin 100 µg/mL).

### 2.2. Plasmid Construction

PCR reactions were carried out in a total volume of 50 µL using, 10 ng of pNZ2122 (MoBiTec), primers pairs listed in Table 1 and Phusion High-Fidelity DNA Polymerase (Thermo Fisher Scientific, Waltham, MA), following the manufacturer’s directions.

The amplicon obtained with the pNZ2122_680F/pNZ2122_395R primers pair (Table 1) was digested with KpnI (NEB), self-ligated using T4 DNA Ligase (NEB) and transformed in *L. lactis* NZ3000. The resulting plasmid, named pTN, was the template for a second PCR using the primers pair pNZ2122_803ClaIF/pNZ2122_760SpeIR that, after ClaI/SpeI double digestion was ligated with the Km^R^ cassette.

The Km^R^ gene cassette was amplified from pKD13, using the pKD13_P1_ClaI/pKD13_P4_SpeI (derived from P1 and P4 primers described by Datsenko and Wanner [34]). The sequence corresponding to P4 was modified in order to mutagenize three restriction sites (namely BamHI, SalI, PstI). The resulting amplicon, after ClaI/SpeI double digestion, was ligated to pTN, previously digested with the same enzymes.

*E. coli* K38 chemically competent cells mixture were transformed with the ligation mixture and plated onto LB Agar plates, supplemented with kanamycin, and incubated overnight at 37 °C.

The sequence of the resulting vector, named pTN-Switch, was verified by Sanger sequencing (Macrogen, Seoul, South Korea).

For a complete list of plasmids used in this study, see Table 2.

DNA for PCR analyses on single colonies was obtained using a direct lysis method [48].

### 2.3. Cassette Removal Tests

pCP20 was transformed into K38 cells containing pTN-Switch and transformants were selected onto LB Agar plates supplemented with kanamycin and ampicillin at 30 °C.

Liquid cultures from single colonies were grown at 30 °C up to OD_600_ 0.7, then shifted to 42 °C so as to induce the expression of FLP recombinase and the block of pCP20 replication. Aliquots were withdrawn at 45, 90, and 120 min, and the plasmid extracted and visualized by agarose gel electrophoresis.

Cells were also diluted and plated on both LB Agar plates containing kanamycin or no antibiotic, and incubated overnight at 42 °C.

The resulting plasmid mix was digested with PstI and transformed into *L. lactis* NZ3000 (following the protocol suggested by the strain provider, MoBiTec), which was selected onto Elliker medium (20 g/L Tryptone 5 g/L, Yeast extract 4 g/L, Sodium chloride 1.5 g/L, Sodium acetate 0.5 g/L, l-(+) Ascorbic acid, 15 g/L agar, 0.5% lactose).

NZ3000/pNT-Switch(ΔKm^R^) colonies were screened by PCR using the pKD13_P1/pKD13_P41 primers pair.

## 3. Results and Discussions

The food-grade expression vector pNZ2122, designed for constitutive, lacA-driven expression of recombinant proteins in *L. lactis*, was chosen as backbone plasmid for subsequent modifications and improvements. Regulatory elements responsible for the host range, plasmid replication, maintenance, and selection, as well those involved in protein expression were not altered, so as to keep the overall expression apparatus unchanged. In fact, the performances of the expression system are well established, but the presence of a long polylinker and the lack of additional elements suitable for subsequent steps of protein purification, prompted us to replace the MCS region with a new one.

In particular, pNZ2122 was amplified using specifically-designed primers (namely pNZ2122_680F/pNZ2122_395R) so as to obtain amplicons consisting in a linear molecule where the original polylinker was replaced by the new one, harboring EcoRI, SacI, SalI, XhoI, BamHI, KpnI and SpeI, followed by a thrombin cleavage site and sequence encoding a 6xHis Tag.

Such arrays ensure easy cloning, a few extra aminoacidic residues even when internal restriction sites are exploited for cloning, the efficient purification of recombinant proteins by IMAC (Immobilized Metal Affinity Chromatography) and the removal of such tag by simple thrombin cleavage. Moreover, the location of the latter element at the C-term in the corresponding protein allows for the selective recovery of full-length products, thus avoiding incomplete molecules.

After circularization, the resulting plasmid, named pTN, was transformed into *L. lactis* NZ3000.

However, as the drawbacks of cloning in *L. lactis* remained, we made efforts in order to overcome such limitation, so as to extend its host range to *E. coli*, without compromise the food-grade status of the final expressing clones.

Taking advantage on the broad host range of the replication origin of pNZ vectors series, where food grade ones cannot be maintained in *E. coli* because of the lack of selective markers, we decided to introduce a removable Km^R^ cassette, flanked by FRT sites.

**Figure 1:**
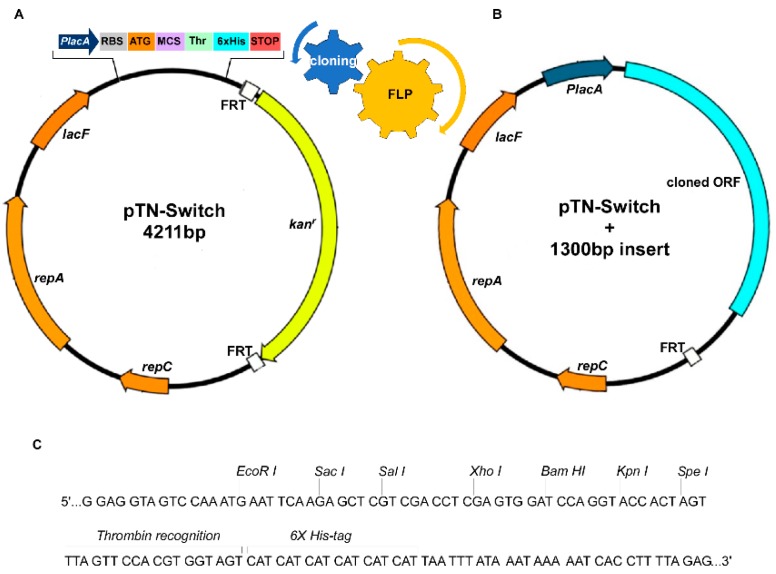
A map of pTN-Switch vector for improved cloning and food grade production. (**A**) The map shows the features of pTN-Switch including the array of functional elements in the MCS and the removable Km^R^ cassette, flanked by FRT sites; (**B**) map of recombinant pTN-Switch after cloning a DNA fragment and Km^R^ cassette excision by flippase; (**C**) the MCS of pTN-Switch vector enabling easy cloning, expression, and purification of recombinant proteins.

The resulting vector, named pTN-Switch (Figure 1A–C), can be transformed and maintained in recA^+^ *E. coli* strains under antibiotic selection. In the same strains all cloning and manipulation steps can be carried out, without limitations otherwise imposed by Gram-positive hosts.

Because of the presence of the antibiotic resistance marker, pTN-Switch cannot be considered as food-grade; however, it can switch to the food-grade status after proper KmR cassette excision, mediated by FLP recombinase expressed from the pCP20 plasmid [45].

The KmR cassette loss generates the food-grade plasmid pNT-Switch(ΔKmR) (Figure 1B).

**Figure 2:**
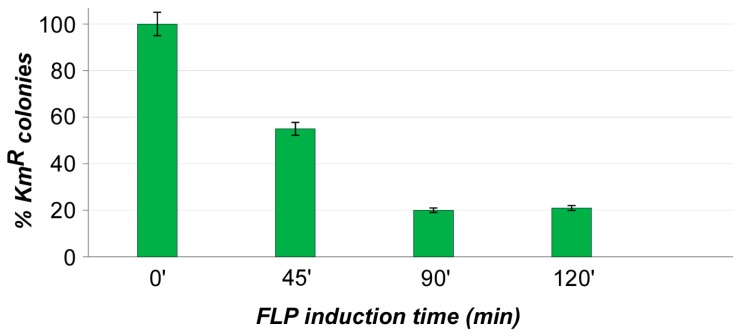
FLP-mediated KmR cassette excision. The resistance cassette loss was measured over time after FLP induction. The percentage was calculated as KmR/total plate count.

In order to assess the efficiency of KmR cassette removal, we tested different times of FLP recombinase action. Exponentially growing cells of *E. coli* K38/pTN-Switch/pCP20 were shifted at 42 °C (FLP induction temperature) and withdrawn at various times after induction. Plasmid was extracted and gel analyzed, whereas aliquots of cells were plated onto proper media (see materials and methods). Electrophoretic analyses showed apparent bands corresponding to pNT-Switch(ΔKmR); plates showed a proportion of kanamycin resistant cells decreasing over time, although such assay results in an underestimation of excision events, as even single plasmid molecule escaped to FLP action can restore the kanamycin resistance. However, both tests, taken together, made us able to evaluate the trend of cassette loss (Figure 2).

The best results were obtained with 90 minutes of induction, as longer incubation times did not result in significant improvement in excision efficiency. As this is in contrast with theoretical expectations, we hypothesized that probably the pTN-Switch replication and the cell division rate, along with the block of pCP20 replication (which impairs plasmid segregation) and the low FLP recombinase cytoplasmic concentration, promotes the pTN-Switch integrity rather than the Km^R^ cassette loss.

In details, the entire workflow includes cloning steps and selection in *E. coli* K38 (or other recA^+^ strains), followed by transformation with pCP20 and thermal induction of FLP recombinase from liquid cultures. The latter step allows the efficient loss of Km^R^ cassette, thus restoring the food-grade structure of the plasmid. The resulting pNT-Switch(ΔKm^R^) is recovered from *E. coli* K38 (with the typical yield of low copy number plasmids of about 700 ng DNA/ml culture) and transformed into the final Gram-positive host, where food-grade expression will actually occur. Transformation efficiency in *L. lactis* was unchanged compared to that of the parental vector, as expected.

As the plasmid extracted after FLP induction is inherently a mixture of pNT-Switch and pNT-Switch(ΔKm^R^), in order to further improve the selective transformation of *L. lactis* NZ3000 with the food-grade plasmid, a unique PstI restriction site located within the Km^R^ ORF can be exploited for a digestion step after extraction. The pNT-Switch linearization results in a dramatic reduction of its transforming ability. It is evident that such treatment requires a careful knowledge of the restriction map of the cloned gene, or a proper sequence optimization aiming to avoid internal PstI sites, so as to allow to exploit such strategy.

A PCR screening of recombinant NZ3000/pNT-Switch(ΔKm^R^) colonies, using the pKD13_P1/pKD13_P41 primers pair, can be carried out, and is strongly suggested in order to check for the actual loss of the antibiotic resistance gene.

## 4. Conclusions

The new expression vector described herein, based on a well-established tool widely used in the field of molecular genetics of microorganisms, offers enormous advantages for food-grade cloning compared to existing and approved procedures. In fact, besides improvements dealing with gene cloning and proteins purification, the most innovative feature consists in the possibility of switching from a vector able to be selected in *E. coli* thanks to a resistance marker to a molecule devoid of any potentially threatening sequence (i.e., Km^R^) to be transferred into a GRAS host, to achieve food-grade expression.

Nonetheless, further improvements and strategies may be hypothesized and might be worthy to be tested.

With regards to the cloning technique, a vector where cloning does not rely on restriction enzymes, like LIC (Ligase Independent Cloning), may be useful. Such an approach, even if less efficient than that based on restriction enzymes and in vitro ligation, has several advantages, including the low cost and the possibility of using the same vector preparation for any in-frame cloning. A vector suitable for such an approach may be achieved by simply replacing the MCS with proper sequences through a PCR amplification (using pTN-Switch as a template) with properly 5’-tailed primers, followed by T4 DNA Polymerase treatment.

Other improvements may deal with the *L. lactis* NZ3000 recombinants selection on lactose, which relies on the sugar utilization efficiency. The complementation of the chromosomal gene deletion is based on the plasmid-borne *lacF* copy, whose expression might be improved (for example through codon optimization, as well as replacing the GTG start codon with the more efficient ATG), so as to overcome the slow-growing phenotype commonly experienced with such a selection. Moreover, especially in vectors for constitutive expression, including integrative ones, clone selection, and maintenance based on the actual expression of the cloned gene might be useful so as to overcome the problem of the recombinant construction instability. As strategies based on translational coupling between the cloned gene and a selective marker proved to be suitable in other systems [49,50], the same tool may be worthy to be tested in *L. lactis* vectors as well, including food-grade ones, where an optimized *lacF* might be located downstream of the cloning site and translationally coupled to the recombinant gene. Such a strategy would ensure the negative selection of cells which have undergone rearrangements affecting regulatory regions involved in the recombinant gene expression.

Overall, such strategies might greatly improve the performances of the cloning and expression systems in GRAS microorganisms.

## Figures and Tables

**Table 1:** The PCR primers list.

Primer Name	Sequence (5′-3′)
pNZ2122_680F	CAGGTACCACTAGTTTAGTTCCACGTGGTAGTCATCATCATCATCATCATTAATTTATAAATAAAAATCACCTTTTAGAG
pNZ2122_395R	AGTGGTACCTGGATCCACTCGAGGTCGACGAGCTCTTGAATTCATTTGGACTACCTCCTAAAT
pNZ2122/803ClaF	TCAAATCGATTCCACCAATTAAAGGACCGATAAC
pNZ2122/760SpeR	TCAACTAGTATTCTGCTCCCGCCCTTATG
pKD13_P1Cla	TCAAATCGATGTGTAGGCTGGAGCTGCTTC
pKD13_P4Spe	TCAACTAGTGAATTAATTCCGGAGATCCATCGACGTGCAGTTC
pKD13_P1	GTGTAGGCTGGAGCTGCTTC
pKD13_P41	GAGATCCATCGACGTGCAGTTC

**Table 2:** The plasmids list.

Plasmid	Markers	Reference
pNZ2122	*lacF*	[37]
pTN	*lacF*, new MCS, Thrombin CS, 6xHis-Tag	This Study
pTN-Switch	pTN, FRT-Km^R^-FRT	This Study
pKD13	bla, FRT, Km^R^	[46]
pCP20	[cI857](λ)(ts),bla, cat, FLP, repA101(ts)	[45]
